# Cytotoxic Activity of Thirteen Endemic and Rare Plants from Chaharmahal and Bakhtiari Province in Iran

**DOI:** 10.22037/ijpr.2019.14665.12599

**Published:** 2019

**Authors:** Seyed Ahmad Emami, Shima Kheshami, Elham Ramazani, Maryam Akaberi, Milad Iranshahy, Seyed Mahammad Kazemi, Zahra Tayarani‐Najaran

**Affiliations:** a *Department of Traditional Pharmacy, School of Pharmacy, Mashhad University of Medical Sciences, Mashhad, Iran. *; b *Department of Biology, Faculty of Science, Ferdowsi University of Mashhad, Mashhad, Iran.*; c *Medical Toxicology Research Center, Mashhad University of Medical Sciences, Mashhad, Iran.*; d *Biotechnology Research Center, Mashhad University of Medical Sciences, Mashhad, Iran.*

**Keywords:** Achillea, Nepeta, Phlomis, Scutellaria, Tanacetum, Cytotoxic, Apoptosis

## Abstract

Chaharmahal and Bakhtiari Province is one of the most important endemism states of the flora of Iran with a considerable plant species diversity. In the present study, the cytotoxic activity of 13 plant species grown in Chaharmahal and Bakhtiari have been evaluated on prostate (PC-3), breast (MCF-7), liver (HepG2), ovary (CHO), and melanoma (B16-F10) cancer cell lines. The cytotoxicity and apoptotic activity of methanol extracts was evaluated using resazurin reagent and flow cytometry of PI stained cells, respectively. Methanol extracts of *Dionysia sawyeri*, *Stachys obtusicrena *and *Cicer oxyodon* on CHO cell line (*p* <0.05) and *D. sawyer* and *Linum album* on B16/F10 cell line (*p *<0.05) showed significant cytotoxic effects and increased apoptosis. It is generally suggested that the plant extracts with low IC_50_ values are likely to be used as anti-cancer compounds in reducing cancer progression in scientific studies.

## Introduction

Cancer is the most recognized term used for more than 100 different types of malignancies that can affect the body (1). The number of various cancer deaths in 2015 was 8.8 million people which is equivalent 1 in 6 worldwide death cases (2). One of the common problems in treating cancers is the tumor cells resistant to conventional chemotherapeutic drugs. This phenomenon leads to the formation of the cells with more aggressive phenotype which are more likely to metastases to other tissues (3). Additional to the mentioned problems in treating cancer, side effects of chemotherapy also attract researchers to investigate new approaches in cancer treatments such as the use of natural resources as therapeutic compounds (4). 

In the present study, we have chosen 13 different endemic and rare plant species from Chaharmahal and Bakhtiari Province, Iran, where there is not any report about the cytotoxic activity of the plants. The plants from the same genus have some similarities in the case of phytochemicals and consequently in their biologic activity, therefore the results of studies on the same species were discussed. Lu and colleagues (2016) reported that the phlomisoside (a diterpenoid) from *Phlomis younghusbandii* (Lamiaceae) have a significant inhibitory effect on the growth, proliferation, migration, and invasive properties of A549 (human lung cancer cells) cancer cell line with IC_50_ of 54.51 μM and induces apoptosis (5). Ji and his colleagues (2015) evaluated cytotoxic activity of *Scutellaria baicalensis* on HepG2 (human liver cancer cells), SW480 (Human colon cancer cells), and MCF-7 (human breast adenocarcinoma cell) cancer cell lines. The results demonstrated that most of the flavones exhibited a significant cytotoxic effect (6). Tayarani-Najaran *et al*. (2010) addressed the cytotoxic effects of total methanol extract and different fractions of *Scutellaria lindbergii* on AGS (Human stomach cancer cells), HeLa (Human cervix cancer cells), MCF-7, and PC12 (rat adrenal gland cancer cells) cells. Based on the results, methylene chloride fraction has shown the most potent cytotoxic activity among the other fractions and decreased cell viability (7).* Scutellaria pinnatifida *and its active component neobaicalein (skullcapﬂavone II) and wogonin showed strong cytotoxic activity against HL-60 and K562 leukemic cell lines (8).

Due to specific climatic conditions and the high diversity of plant species in different regions of Iran, investigation of the therapeutic properties and molecular functional mechanisms of the rare plant species to explore new drugs for treating various diseases, including cancer is worthy. One of the rich areas with diverse plant species in Iran is Chaharmahal and Bakhtiari Province. Chaharmahal and Bakhtiari Province is located in the middle of the mountains of the west of Iran and the plain of Isfahan. The situated area is located between 31 14` and 33 47` N (latitude), 49 49` and 51 34` E (longitude). About 1400000 hectares of the total area of the Province which is equivalent to 86.6% of all total area is occupied with forests and pastures (9).

In this study cytotoxic effects and apoptosis induction of methanol extract from thirteen rare plants from Chaharmahal and Bakhtiari Province, including species from the families listed in Table 1, were assessed on the human prostate cancer (PC-3), (MCF-7), (HepG2), Chinese hamster ovary cells (CHO), and murine melanoma (B16-F10) cancer cell lines. To the best of our knowledge, this is the first report on cytotoxic activity of the plants.

## Experimental

*Plant materials*

Thirteen species of the endemic and rare plants from different families were collected in spring and summer 2015 from various regions of Chaharmahal and Bakhtiari Province, southwestern of Iran and identified by Dr. S. H. A. Shirmardi (Table 1). Voucher specimens of the species were deposited in the herbarium of School of Pharmacy, Mashhad University of Medical Sciences, Mashhad, Iran. 

*Preparation of extracts*

The aerial parts of each species were dried in shadow and powdered. Then, 100g of each powder was macerated with methanol for 24 hr at controlled room temperature (25 °C). The macerated powder sample was percolated using pure methanol. Then the methanol extracts were concentrated via a rotary evaporator under the reduced pressure at 50 ˚C and subsequently freeze dried. All extracts were stored at -20 °C. The yield percentage of the obtained extracts were presented in Table 2.

*Cell culture and treatment*

The prostate (PC-3), breast (MCF-7), liver (Hep G2), ovary (CHO), and melanoma (B16/F10) cancer cell lines (code numbers: C427, C135, C158, C111, and C540) were obtained from Cell Bank at the Pasteur Institute (Tehran, Iran). CHO were cultured in F-12K medium (Sigma) with 10% (v/v) fetal bovine serum, 100U/mL penicillin and 100 mg/mL streptomycin. Cell lines B16 F10 and MCF7 were cultured in Dulbecco›s Modified Eagle›s Medium (DMEM) (Sigma) with 10% (v/v) fetal bovine serum, 100U/mL penicillin and 100 mg/mL streptomycin, while the other cell lines were cultured in RPMI 1640 medium (Sigma) with 10% (v/v) fetal bovine serum, 100U/mL penicillin, and 100 mg/mL streptomycin. Then all of the cells were kept at 37 °C in a humidified atmosphere (90%) containing 5% CO_2_. For each concentration and time course study, there was a control sample, which remained untreated and received an equal volume of the solvent (22). 

*Cell viability*

The resazurin reagent is a cell viability indicator that allows measuring the cytotoxicity of various chemical components. For detection of the cell viability, all of cancer cell lines (104 cells per well) were seeded in 96-well plates and were incubated with the methanol extract of each species (50 and 100 μM) for 48 h. Then resazurin reagent (20 μM) was added to each well and incubated for 4 h. The cell viability was assessed at the absorbance of 600 nm with ELISA microplate reader (Awareness, Palm City, FL, USA) (23).

*Flow cytometry analysis of apoptosis*

Flow cytometry and PI staining of the treated cells to detect a sub-G1 peak evaluated Apoptotic cells. CHO cells (105 cells per well) were cultured into 24-well plates and were treated with the methanol extract of *D. sawyeri*, *S. obtusicrena, *and *C. oxyodon *(50 and 100 μM) for 48 h and also B16/F10 were treated with the methanol extract of for 48 h. the cells were washed with phosphate-buffer saline (PBS). After trypsinization, the cells were harvested and incubated at 4 °C in dark with 400 μL of hypotonic buffer (50 μg/mL PI in 0.1% sodium citrate and 0.1% Triton X-100) for 30 s before flow cytometric analysis using a Partec flow cytometer (GmbH, Münster, Germany) 

(23).

*Statistics analysis*

One-way analysis of variance (ANOVA) and Turkeys-Kramer post hoc were used for data analysis. All of the results were expressed as mean ± SD and p-values below<0.05 was regarded statistically significant.

## Results

**Figure 1:**
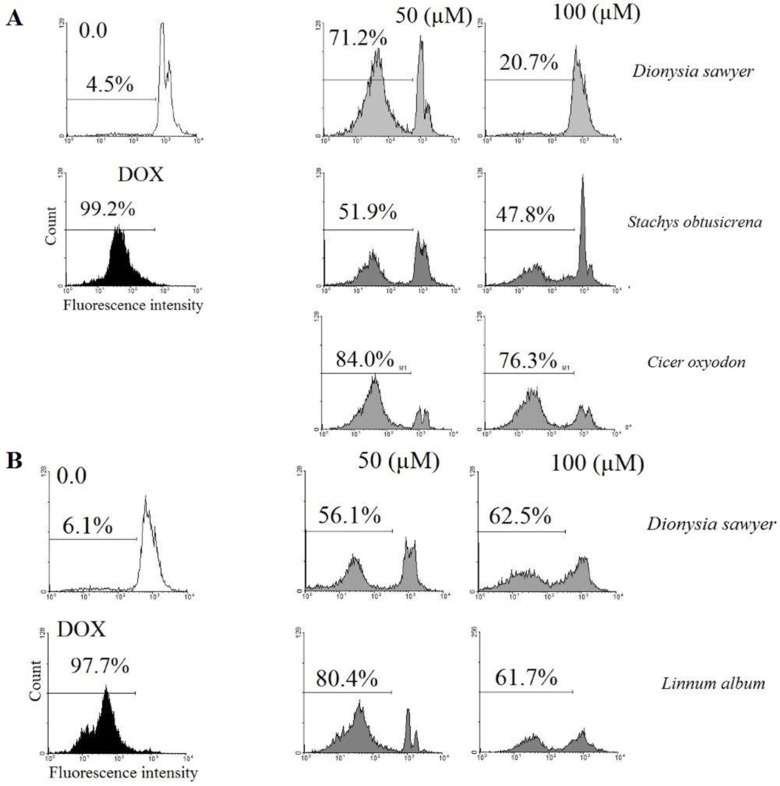
Flow cytometry histograms of apoptosis assays by PI method of CHO and B16/F10 cells: (A) CHO cells were incubated with 50, 100 µM of Methanol extracts of *Dionysia sawyer*, *Stachys obtusicrena *and *Cicer oxyodon *and (B) B16/F10 cells were incubated with 50, 100 µM of D. sawyer and *Linnum album *for 48 h. All of the components induced cell death through apoptosis. All experiments were done in triplicate

Based on the results, the extracts of *D. sawyeri, S. obtusicrena *and* C. oxyodon *on CHO cell line (*p* <0.05) and the extracts of *D. sawyeri* and *L. album* on B16-F10 cell line (*p* <0.05) decreased cell viability and showed significant cytotoxic effects. (Tables 3 and 4). Also, results demonstrated that the extracts of *D. sawyeri*, *S. obtusicrena, *and *C. oxyodon *on CHO cell line induced cell death through apoptosis (Figure 1).

## Discussion

Variation in the plant species as well as the presence of the exclusive plant species, that have not been studied so far require extensive biological screening to find putative natural substances as effective agents for the treatment of the disease. Many substances including natural products and phytochemicals have been screened to find an optimal treatment for cancer as the second cause of death worldwide. 

In this study, the cytotoxic effects of methanol extract from 13 plant species from Chaharmahal and Bakhtiari Province were investigated on PC-3, CHO, B16/F10, HepG2 and MCF-7 cancer cell lines. According to the results, methanol extract of *D. sawyeri*, *S. obtusicrena, *and *C. oxyodon *in CHO cells and methanol extract of *D. sawyer* and *L. album *in the B16/F10 cells decreased cell viability and showed significant cytotoxic activity. Also methanol extract of *D. sawyeri*, *S. obtusicrena, *and *C. oxyodon *increased apoptosis induction in CHO cells. This is the first study that evaluated the cytotoxic effects of the rare plants from Chaharmahal and Bakhtiari Province. Regarding the novelty of the present study, there is not any similar evaluation on the species investigated here. Since the plants belonging to the same genus have similarities in presence of alike phytochemicals, we have searched for some evidences of cytotoxicity in similar species in the same genus.

*Dionysia termeana* is one of the similar species to *D. sawyeri*, inhibiting the growth and proliferation of leukemia (K562) and lung carcinoma (A549) cell line with an IC50 less than 20 μg/mL by MTT staining and flow cytometry analyses (24). *Dionysia termeana*, also significantly inhibited the growth and proliferation of lymphocyte cells (25). In our study, *D. sawyeri* exerted a cytotoxic effect through decreasing cell viability and increasing amount of apoptosis. 

There are many reports on the cytotoxic activity of the plants belonging to the genus *Stachys*. Jassbi *et al*. (2014) examined the cytotoxic effects of methanol and dichloromethane extracts of nine different species of *Stachys* on HL-60, K562, and MCF-7 cancer cells. The authors reported that dichloromethane extract of *S. pilifera* had the lowest IC50 on HL-60 (Human leukemia cancer cells), K562 (Human leukemia cancer cells), and MCF-7 cancer cells (ranging from 33.1 to 18.4 μg/mL) (26). In another study, it has been shown that *S. alopecuros* inhibit the growth of A375 (Human melanoma cancer cells), HCT116 (Human colon cancer cells), and MDA-MB 231 (Human breast cancer cells) cells with IC_50_ less than 20 μg/mL (27). Also, the volatile oil of *S. rupestris* has been shown to inhibit the growth and proliferation of PC-3 and MCF-7 cell lines (28). In our study, *S. obtusicrena *showed inducing an effect on apoptosis and cell growth inhibition.

*Cicer microphylluma* similar species from the same genus of *C. oxyodon*, has shown potent cytotoxic activity against mammary melanoma cell lines and human epidermis carcinoma (29). The activity was attributed to the presence of luteolin in the plant (29). Isoflavones extracted from *C. arietinum* promoted the growth of MCF-7 cell line at low concentrations and inhibited the growth and proliferation of the cells at high concentrations (more than 1 mg/L) (30). Isoflavones extracted from the *C. arietinum* inhibited the growth and proliferation of two human breast cancer cell lines including SKBR3 (Human breast cancer cells) and MCF-7 (31). 

*Dionysia sawyeri, S. obtusicrena*, *C. oxyodon, *and *L. album *as the most cytotoxic and CHO and B16/F10 cells as the most sensitive cells were chosen for the future mechanistic activity. In our study, *D. sawyeri*, *S. obtusicrena*, and *C. oxyodon *increased apoptosis induction which was confirmed after PI staining of the cells and flow cytometry analysis on CHO cells. In the present study for the first time, the cytotoxic activity of *L. album* was reported. *L. album* caused a dose-dependent cytotoxic activity on the B16-F10 cell line with minimal effect on other cells.

**Table 1:** Medicinal plants evaluated for cytotoxic activity from Chaharmahal and Bakhtiari Province of Iran

**Species**	**State**	**Family**	**Voucher number**	**Location**
*Achillea kellalensis *Boiss. & Hausskn.	endemic	Asteraceae	13206	Gelougerd, the Northern slopes of mountain Kalar
*Ajuga chamaecistus *Ging. ex Benth.	rare	Lamiaceae	13201	Shahrekord, the mountain Farhangian
*Aristolochia olivieri *Colleg. ex Boiss.	endemic	Aristolochiaceae	13202	Malkhalifeh, Shirmard village
*Cicer oxyodon *Boiss. & Hohen.	rare	Fabaceae	13207	Malkhalifeh, Shirmard village
*Dianthus orientalis *Adams	rare	Caryophyllaceae	13208	Hafshejan, Jouneghan
*Dionysia sawyeri *(Watt) Wendelbo	endemic	Primulaceae	13205	Malkhalifeh, Shirmard village
*Linum album *Kotschy ex Boiss.	endemic	Linaceae	13204	Shahrekord, the mountain Farhangian
*Nepeta glomerulosa *Boiss.	endemic	Lamiaceae	13200	Shahrekord, castle Gharak
*Phlomis aucheri *Boiss.	endemic	Lamiaceae	13199	Shahrekord, castle Gharak
*Picris strigosa *M. Bieb.	rare	Asteraceae	13203	Malkhalifeh, Shirmard village *Scutellaria multicaulis*
Boiss.	endemic	Lamiaceae	13198	Malkhalifeh, Shirmard village
*Stachys obtusicrena *Boiss.	endemic	Lamiaceae	13196	Avargan, the mountain Kalar
*Tanacetum dumosum *Boiss.	endemic	Asteraceae	13197	Malkhalifeh, Shirmard village

**Table 2:** The extraction yield % of medicinal plants

**Species**	**Extraction yield %**
*Achillea kellalensis*	11.77%
*Ajuga chamaecistus*	16.44%
*Aristolochia olivieri*	19.25%
*Cicer oxyodon*	16.46%
*Diantus orientalis*	8.31%
*Dionysia sawyeri*	1.84%
*Linnum album*	14.6%
*Nepate glomerulosa*	7.6%
*Phlomis aucheria*	16%
*Picris strigosa*	4.4%
*Scutellaria multicauli*	7.41%
*Stachys obtusicrena*	17.01%
*Tanaetum dumosum*	11.37%

**Table 3:** Biological properties and chemical constituents of medicinal plants from Chaharmahal and Bakhtiari Province of Iran

**Species**	**Chemical constituents**	**Biological activities**
*Achillea kellalensis*	Camphor (34.0%), borneol (12.6%), β-thujone (12.5%), 1,8-cineole (11.3%), bornyl acetate (7.3%), camphene (7.0%) (10)	Antioxidantand, antibacterial (11)
*Ajuga chamaecistus*	Melilotoside, phenylethyl glycosides, phytoecdysteroids (12)	Antidiabetic (13), anti-inflammatory (14), antibacterial (15)
*Aristolochia olivieri*	-	-
*Cicer oxyodon*	-	-
*Dianthus orientalis*	-	-
*Dionysia sawyeri*	-	-
*Linum album*	Podophyllotoxin, 5- methoxypodophyllotoxin (Smollny *et al*., 1998)	Antitumor (16)
*Nepeta glomerulosa*	Geranyl acetate (17.0%), limonene (12.0%), eucalypo (5.8%), bornyl acetate (5.3%), citronellal (4.9%), spathulanol (4.2%), sabinene (3.9%), β-ocimene (3.9%), β-sesquiphellandrene (2.8%), neryl acetate (2.5%), α-humulene (2.4%), α-pinene (2.3%), humulene oxide (2.2%), norsolanadione (2.1%), terpinen-4-ol (2.0%) (17)	Antibacterial (17)
*Picris strigosa*	-	-
*Scutellaria multicaulis*	Trans-caryophyllene (34.6%), caryophyllene oxide (12.2%), linalool (10.7%), germacrene D (5.5%) (Asadollahzadeh and Rajaie, 2014)	Antioxidant
*Stachys obtusicrena*	α-pinene (34.6%), germacrene D (8.0%), bicyclogermacrene (7.8%) (18)	Antibacterial (19), Anti-inflammatory (Amirghofran, 2010), Antimicrobial (20)
*Tanacetum dumosum*	Borneol (27.9%), bornyl acetate (18.4%), 1,8-cineol (17.5%), α-terpineol (5.3%), cis-chrysanthenyl acetate (3.3%), camphene (2.7%), terpinene-4-ol (1.9%) (21)	-

**Table 4:** Cytotoxicity (% of viability) of methanol extract of medicinal plants from Chaharmahal and Bakhtiari Province of Iran

	Concentration^1^														
**Cell line→**	PC3			MCF7			HepG2			CHO			B16-F10		
**Plant name↓**	**0**	**50**	**100**	**0**	**50**	**100**	**0**	**50**	**100**	**0**	**50**	**100**	**0**	**50**	**100**
*Achillea kellalensis*	100.0±19.3	107.0±29.0	104.3±33.2	100.0±39.7	99.7±27.6	110.2±45.5	99.9±13.5	87.5±12.9	93.6±10.5	100.0±39.3	72.1±23.5	77.7±19.5	100.0±7.0	110.6±8.4	88.7±19.1
*Ajuga chamaecistus*	100.0±19.3	105.4±33.3	100.8±41.5	100.0±39.7	105.9±31.5	96.8±38.8	99.9±13.5	103.5±9.3	98.0±11.3	100.0±39.3	66.7±15.7	54.0±8.9	100.0±7.0	102.0±15.2	99.9±8.9
*Aristolochia olivieri*	100.0±19.3	105.7±21.0	111.0±19.7	100.0±39.7	111.3±9.8	121.4±5.6	99.9±13.5	108.1±9.2	112.0±6.1	100.0±39.3	90.1±20.6	79.2±30.6	100.0±7.0	98.5±13.8	108.8±45.1
*Cicer oxyodon*	100.0±19.3	107.7±39.5	101.5±22.9	100.0±39.7	101.0±36.3	96.8±37.5	99.9±13.5	113.0±6.6	120.6±8.4	100.0±39.3	63.3±20.7*	68.5±28.2*	100.0±7.0	81.0±7.8	71.7±10.1
*Dianthus orientalis*	100.0±19.3	107.6±22.7	105.8±23.6	100.0±39.7	104.9±26.1	97.5±39.2	99.9±13.5	89.4±9.5	95.4±10.9	100.0±39.3	73.4±23.0	79.7±24.6	100.0±7.0	81.0±30.6	102.3±18.4
*Dionysia sawyeri*	100.0±19.3	95.3±27.8	98.6±29.0	100.0±39.7	91.2±13.7	104.6±14.4	99.9±13.5	93.0±7.2	87.3±6.0	100.0±39.3	62.3±16.8*	58.6±19.7*	100.0±7.0	76.5±21.3*	62.9±12.8*
Doxorubicin	100.0±19.3	58.6±18.7*	46.6±17.1*	100.0±39.7	36.4±26.8**	52.7±21.1*	99.9±13.5	38.8±15.2**	40.0±15.2*	100.0±39.3	64.8±26.3*	71.2±15.7*	100.0±7.0	20.4±12.5**	36.2±11.7**
*Linum album*	100.0±19.3	110.8±20.7	100.9±15.1	100.0±39.7	120.3±21.4	120.0±13.3	99.9±13.5	96.2±10.2	97.7±13.6	100.0±39.3	88.1±16.8	74.7±11.7	100.0±7.0	63.6±6.8*	69.6±20.1*
*Nepeta glomerulosa*	100.0±19.3	102.4±20.4	106.1±19.5	100.0±39.7	125.9±13.7	117.8±12.9	99.9±13.5	96.7±10.3	101.5±8.0	100.0±39.3	88.9±29.4	86.5±28.3	100.0±7.0	102.5±10.4	111.8±13.8
*Phlomis aucheri*	100.0±19.3	103.8±27.0	97.8±12.8	100.0±39.7	112.7±12.5	116.7±16.6	99.9±13.5	89.7±10.0	97.6±11.2	100.0±39.3	98.2±19.9	75.8±24.6	100.0±7.0	104.0±16.9	96.3±21.0
*Picris strigosa*	100.0±19.3	106.4±23.0	104.5±36.0	100.0±39.7	114.0±11.1	112.0±11.1	99.9±13.5	102.5±8.9	105.1±5.9	100.0±39.3	70.8±17.8	76.0±18.4	100.0±7.0	114.5±9.5	92.9±21.9
*Scutellaria multicaulis*	100.0±19.3	96.8±25.2	96.7±36.1	100.0±39.7	104.8±26.1	91.9±5.8	99.9±13.5	92.5±8.6	94.8±4.2	100.0±39.3	58.5±43.9	72.6±42.0	100.0±7.0	89.3±16.5	79.5±20.8
*Stachys obtusicrena*	100.0±19.3	120.2±18.2	98.3±21.0	100.0±39.7	102.8±25.7	109.8±10.4	99.9±13.5	90.2±12.8	87.5±5.3	100.0±39.3	61.1±20.0*	53.7±6.3*	100.0±7.0	86.7±21.3	98.7±17.7
*Tanacetum dumosum*	100.0±19.3	91.6±29.8	92.2±28.2	100.0±39.7	79.3±35.4	100.0±14.8	99.9±13.5	99.7±7.8	112.3±8.3	100.0±39.3	89.9±29.8	78.8±32.3	100.0±7.0	87.0±25.9	74.2±38.2

^1^ The concentration of extracts is presented in µg/mL.

Dunnet's Multiple Comparison Test;*p < 0.05, **p < 0.001

## Conclusion

Two common types of cancer in young male and female are melanoma and ovarian cancer. Melanoma is one of the most hazardous forms of skin cancer, which occurred in 232,000 people and resulted in 55,000 deaths in 2012. Surgery is mostly used to treat this cancer by removing involved parts. Ovarian cancer is the seventh most common cancer in women and the eighth leading cause of cancer death in the world. This occurred around 239,000 cases and resulted in 152,000 deaths worldwide in 2012. Treatment usually involves a combination of surgery, radiation therapy, and chemotherapy. 

Among four cytotoxic plants introduced in this study, the main constituents from *S. obtusicrena *and *L. album *have been reported in table 3. Based on the diversity of the chemicals present in these plants, it is suggested that further analytical and mechanistic evaluation supports the use of the plant as potential anticancer agents.
